# Blackwater Fever in Ugandan Children With Severe Anemia is Associated With Poor Postdischarge Outcomes: A Prospective Cohort Study

**DOI:** 10.1093/cid/ciz648

**Published:** 2019-07-12

**Authors:** Robert O Opoka, Ali Waiswa, Nambuya Harriet, Chandy C John, James K Tumwine, Charles Karamagi

**Affiliations:** 1 Department of Pediatrics and Child Health, College of Health Sciences, Makerere University, Kampala; 2 Global Health Uganda (GHU) Research Collaboration, Kampala; 3 Nalufenya Children’s Ward, Jinja Regional Referral Hospital, Uganda; 4 Ryan White Center for Pediatric Infectious Disease and Global Health, Indiana University School of Medicine, Indianapolis

**Keywords:** blackwater fever, severe anemia, postdischarge, mortality

## Abstract

**Background:**

Blackwater fever (BWF), one of the complications of severe malaria, has recently re-emerged as a cause of severe anemia (SA) in African children. However, postdischarge morbidity in children with BWF has previously not been described.

**Methods:**

This was a descriptive cohort study in which children, aged 0–5 years, admitted to Jinja Regional Referral Hospital with acute episodes of SA (hemoglobin ≤5.0 g/dL) were followed up for 6 months after hospitalization. Incidence of readmissions or deaths during the follow-up period was compared between SA children with BWF and those without BWF.

**Results:**

A total of 279 children with SA including those with BWF (n = 92) and no BWF (n = 187) were followed for the duration of the study. Overall, 128 (45.9%) of the study participants were readmitted at least once while 22 (7.9%) died during the follow-up period. After adjusting for age, sex, nutritional status, and parasitemia, SA children with BWF had higher risk of readmissions (hazard ratio [HR], 1.68; 95% confidence interval [CI], 1.1–2.5) and a greater risk of death (HR. 3.37; 95% CI, 1.3–8.5) compared with those without BWF. Malaria and recurrence of SA were the most common reasons for readmissions.

**Conclusions:**

There is a high rate of readmissions and deaths in the immediate 6 months after initial hospitalization among SA children in the Jinja hospital. SA children with BWF had increased risk of readmissions and deaths in the postdischarge period. Postdischarge malaria chemoprophylaxis should be considered for SA children living in malaria endemic areas.

## Teaser

There is a high frequency of postdischarge readmissions and deaths in children with severe anemia, especially in severe anemia associated with blackwater fever. Postdischarge malaria prophylaxis should be considered in children with severe anemia.

Severe anemia (SA) (hemoglobin [Hb] <5 g/dL) remains a public health problem in resource-limited settings, accounting for up to 9.7–29% of all pediatric admissions and 8–17% of inpatient deaths [1–4]. *Plasmodium falciparum* malaria is the cause of a severe clinical spectrum of malaria, including SA, in malaria-endemic areas [5, 6]. Several studies have shown that severe malarial anemia is associated with significant morbidity in the immediate postdischarge period [7–9].

Although the cause of SA is multifactorial, malaria infection remains the main driving factor for the high prevalence of SA in sub-Saharan Africa [10, 11]. One manifestation of malaria associated with SA is blackwater fever (BWF), a resurgence of which has recently been reported in parts of East Africa [12]. Blackwater fever is a massive intravascular hemolytic event that is thought to be triggered by malaria infection [13] and use of antimalarials such as quinine [14, 15], mefloquine [16, 17], artemisinins [12], halofantrine [16–18], and/or lumefantrine, which is a second component of artemisinin-based combination therapies [19]. There is a paucity of data on the etiology of BWF in Ugandan children. This cryptic disease typically presents clinically with SA associated with episodes of passage of dark or tea-colored urine [13]. In eastern Uganda, BWF was reported in 14.5% and 21.8%, respectively, of sick children presenting to 2 regional referral hospitals [20]. The few studies on BWF have mainly been hospital based with limited information on the postdischarge outcomes [21–23]. It is not clear whether the postdischarge morbidity of SA associated with BWF and other nonmalarial etiologies is similar to that previously reported. Hence, there are presently no specific guidelines for postdischarge management of SA, even for that associated with malaria. To inform further studies on postdischarge SA there is a need to establish the postdischarge outcomes of SA in areas where the burden of BWF is high.

We followed up a cohort of children aged 0–5 years admitted with SA to a tertiary hospital in Uganda. Our aim was to compare the frequency and risk of readmissions and death among children with SA complicated by BWF with those children with SA without BWF in the immediate 6 months after discharge.

## METHODS

### Design

This was a prospective cohort study carried out at Jinja Regional Referral Hospital (Jinja RRH) in southeastern Uganda. Jinja RRH serves a mainly rural population in a region of high malaria transmission between the shores of Lakes Victoria and Kyoga [24].

### Study Participants

From June 2016 to January 2018, children with SA (Hb ≤5.0 g/dL) aged 0–5 years presenting to the pediatric ward of Jinja RRH were screened for enrollment into the study. Children who had been transfused in the previous 4 weeks or who presented due to surgical conditions or those with SA due to known chronic conditions such as tuberculosis or malignancies were excluded. Children with SA due to sickle cell anemia (confirmed by Hb electrophoresis) were also excluded since their underlying condition is known to be associated with recurrent readmissions and anemia. During hospitalization, enrolled participants were managed according to the routine standard of care as per national treatment guidelines [25], which included a prompt blood transfusion, investigations to establish associated comorbid diagnoses, and treatment of comorbid conditions.

### Tests and Treatment

Malaria microscopy was performed for all children, and children with parasitemia were given parenteral artesunate for at least 24 hours followed by a full course of artemether-lumefantrine as per guidelines [25]. Other tests to establish comorbid diagnoses associated with the SA included a blood culture for bacteremia if the axillary temperature was greater than 38.0^°^C; screening for the human immunodeficiency virus using a statapak kit STAT-PAK (Chembio); urine and stool microscopy for schistosomiasis and worm infestations, respectively; serum assays for levels of ferritin, folate, and vitamin B-12; and complete blood count to type the anemia. As per guidelines, blood transfusion was given to all children with SA with Hb of 5.0 g/dL or less at a dose of 10 mL/kg (packed cells) or 20 mL/kg whole blood. Broad-spectrum antibiotics were given for suspected bacteremia; hookworm infection was treated with oral albendazole. All children were put on a 3-month regimen of oral hematinics on discharge.

The diagnosis of BWF was made on clinical grounds. Any child with a history of passage of dark-brown, tea-colored urine during the present episode of illness observed by the caregiver and, where possible, by the study clinician was diagnosed as having BWF. No clinical tests were done to confirm the diagnosis. Children with BWF were managed symptomatically with blood transfusion and treatment of comorbid conditions. Presently, there is no specific management of BWF, but all cases are treated as severe malaria and the respective supportive treatment such as blood transfusion is given as per the Ugandan clinical guidelines [25].

### Follow-up and Postdischarge Care

On discharge, all study children were asked to return to the hospital for a follow-up visit in 6 months. A predischarge Hb collection was done to ensure that the SA was corrected. Caregivers were also asked to bring their children back to the study team at Jinja RRH whenever the children became sick during the follow-up period. All hospital visits and admissions to Jinja RRH were recorded, and children who presented with a history of fever or had a high temperature on examination had a malaria blood smear and/or rapid diagnostic test performed. At the end of the follow-up period, the vital status of study participants was noted and any deaths that occurred during the follow-up period were recorded.

### Primary Outcome

The primary outcome was a composite of readmissions or deaths within the immediate 6 months after discharge.

### Sample Size

We calculated that a sample size of 279 would give a power of over 90% to detect a 25% difference in frequency readmission or death between SA with and without BWF with a 95% confidence interval (CI) and an ɑ of 0.05. We assumed a death or readmission rate of 12% in the children with SA without BWF [11].

### Statistical Analysis

Data were entered in FileMaker Pro and exported to Stata version 14 for analysis (StataCorp, 2015). Demographic characteristics between the 2 groups were compared using Pearson chi-square and Wilcoxon rank sum tests. The frequency and incidence of study endpoints (outpatient visits, readmissions, or deaths) were determined and compared for children with SA with BWF and those without BWF using chi-square and incident rate ratios, respectively. Hazard ratios (HRs) were calculated and adjusted for age, sex, nutritional status (weight-for-age *z* scores), and parasitemia using Cox regression.

### Ethical Considerations

Ethical approval was granted by the Makerere University School of Medicine Research and Ethics Committee and the Uganda National Council of Science and Technology. Written informed consent was obtained from the caregivers of all the children.

## RESULTS

**Figure 1.**
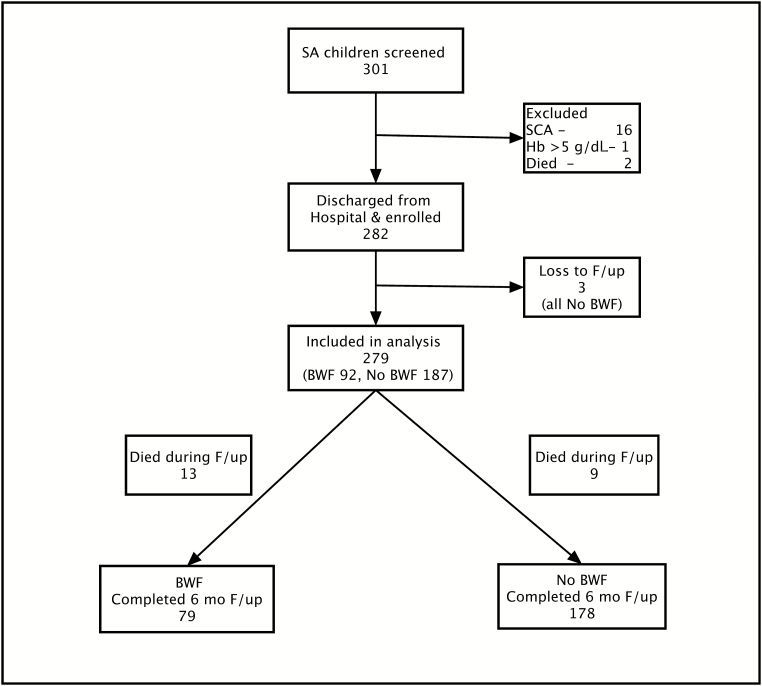
Study profile of children with severe anemia who were screened and followed up. Abbreviations: BWF, blackwater fever; F/up, follow-up; Hb, hemoglobin; SA, severe anemia; SCA, sickle cell anemia.

During the study period, 284 children with SA were admitted to Jinja RRH, of whom 282 were discharged and were eligible for this study (Figure 1). However, 3 children were lost to follow-up and at the end of the 6-month follow-up period, data on vital status (alive or dead) were available for 279 (98.9%) of the eligible children who were included in the analysis (Figure 1). Ninety-two (32.9%) of the children with SA included in the analysis had BWF (Figure 1). There was no difference in the sex distribution, social economic status, and level of predischarge Hb concentration between children with SA with BWF and those without BWF (Table 1). At presentation to hospital, children with SA with BWF were more likely to be older, jaundiced and have a higher concentration of Hb and palpable spleen compared with children SA without BWF (all *P* < .05) (Table 1). However, children with SA without BWF were more likely to be stunted than children with SA with BWF (Table 1). All children in the study received a transfusion at admission but more children with SA with BWF required multiple transfusions during admission than children without BWF (Table 1). At admission, the prevalence of parasitemia and leukocytosis was similar between children with and without BWF (Table 1). The prevalence of exposure to recent malarial infection (positive rapid diagnostic test) was similar between the study groups (Table 1). However, among children with parasitemia at admission, children with SA with BWF had lower mean parasite density than children with SA without BWF (43 826 vs 107 618, respectively; *P* = .04).

**Table 1.** Demographic and Clinical Characteristics of Children With Severe Anemia With and Without Blackwater Fever Discharged From Jinja Regional Referral Hospital and Followed Up for 6 Months

	Children With SA		
	Without BWF(n = 187)	With BWF (n = 92)	*P*
Demographic characteristics			
Mean (SD) age, y	2.2 (1.2)	2.8 (1.0)	.001
Sex, n (% male)	119 (63.6)	57 (61.9)	.785
Mean (SD) total SES score^a^	12.0 (3.7)	11.5 (3.9)	.291
Mean (SD) postdischarge Hb,^b^ g/dL	7.5 (1.2)	7.6 (1.3)	.792
Mean (SD) duration of hospitalization, d	4.2 (2.1)	4.7 (1.7)	.160
Clinical features at presentation			
Referred, n (%)	112 (59.9)	57 (61.9)	.740
Mean (SD) duration of illness, d	3.9 (1.9)	3.6 (1.7)	.226
Previous history of transfusion, n (%)	49 (26.2)	54 (58.7)	<.001
Mean (SD) axillary temperature, °C	37.4 (0.8)	37.3 (0.8)	.340
Stunted (HAZ < −2 SDs), n/N (%)	57/181 (31.5)	15/90 (16.7)	.009
Mean (SD) admission Hb, g/dL)	3.6 (0.9)	3.8 (0.8)	.044
Clinical presentation, n (%)			
Jaundice	28 (14.9)	34 (36.9)	<.001
Hepatomegaly	43 (22.9)	28 (30.4)	.180
Splenomegaly	99 (52.9)	58 (63.0)	.110
Received multiple transfusions, n (%)	71 (37.0)	55 (59.8)	.001
Malaria parasitemia, n (%)	69 (36.9)^c^	35 (38.0)	.852
Recent malarial infection^d^	95/169 (56.2)	45/83 (54.2)	.788
Leukocytosis,^e^ n (%)	55/176 (29.9)	37 (40.2)	.086
HIV positive, n (%)	5 (2.7)	1 (1.1)	.390

Abbreviations: BWF, blackwater fever; HAZ, height-for-age *z* score; Hb, hemoglobin; HIV, human immunodeficiency virus; SD, standard deviation; SA, severe anemia; SES, socioeconomic status.

^a^SES score available for 275 patients (BWF = 91, no BWF = 184).

^b^Hb available for 267 participants (BWF = 90, no BWF = 177).

^c^Malaria parasitemia results available for 91 participants with BWF.

^d^Had positive malarial rapid diagnostic test but no malaria parasitemia at admission.

^e^Leukocytosis = white blood cell count >12.0 /µL.

Overall, 14 (5.0%) of children with SA with malaria parasitemia had other features of severe malaria such as impaired consciousness or respiratory distress. The proportion of children with other features of severe malaria were similar in the study groups.

### Outpatient Clinic Visits, Readmissions, and Deaths During Follow-up

Overall, 72 (25.8%) of study participants had outpatient clinic visits, 128 (45.9%) were readmitted at least once, while 22 (7.9%) died during the follow-up period. The numbers of children who were readmitted or died were significantly higher in children with SA with BWF than in those without BWF: 54.4% versus 41.7% (*P* = .046) and 14.1% versus 4.8% (*P* = .007), respectively (Table 2).

**Table 2.** Six-month Postdischarge Outcomes of 279 Children With Severe Anemia With and Without Blackwater Fever at Initial Admission to Jinja Regional Referral Hospital

	BWF (n = 92)	No BWF(n = 187)	*P*
Children with outpatient clinic visits, n (%)	25 (27.2)	47 (25.1)	.714
Number of outpatient clinic visits	33	63	
Incidence of outpatient clinic visits, events per 100 person-years	74.87	65.85	.274
Children readmitted, n (%)	50 (54.4)	78 (41.7)	.046
Readmissions, no. of events	102	111	
Incidence of readmissions, events per 100 person-years	231.42	116.03	<.001
Deaths, n (%)	13 (14.1)	9 (4.8)	.007
Deaths, no. of events	13	9	
All-cause mortality per 100 person-years	29.49	9.41	.005

Abbreviation: BWF, blackwater fever.

There was a total of 96 clinic visits and 213 readmissions during the study period (Table 2). Children with BWF had a significantly higher incidence of readmissions and deaths than those without BWF: 231.42 versus 116.03 (*P* < .001) and 29.49 versus 9.41 (*P* = .005), respectively (Table 2). The incidence of outpatient visits was also higher in the children with SA with BWF, but the difference was not statistically significant (Table 2).

### Causes of Readmissions

There were multiple admissions in 52 (40.6%) of the readmissions. Children with SA with BWF had more multiple readmissions than those without BWF, with 25 (50%) having more than 1 admission, including 8 (16%) children who had at least 4 readmissions during the 6-month follow-up period (Table 3). Malaria and a recurrence of the SA were the most common reasons for readmissions, accounting for 100 (78.1%) and 47 (36.7%) of the first readmissions and 37 (71.2%) and 21 (40.4%) of the second readmissions, respectively (Table 3). Among the children with SA with BWF, a repeat episode of BWF occurred in 50–60% of the readmissions, while 9 (11.5%) of readmitted children in the no-BWF group reported an episode of BWF at the first readmission (Table 3).

**Table 3.** Postdischarge Readmissions and Their Causes Among 279 Children With Severe Anemia With and Without Blackwater Fever at Jinja Regional Referral Hospital

	Readmission 1		Readmission 2		Readmission 3		Readmission 4	
	No BWF	BWF	No BWF	BWF	No BWF	BWF	No BWF	BWF
Severe malaria^a^	64 (82.1)	36 (72.0)	17 (68.0)	20 (74.1)	5 (71.4)	10 (71.4)	1 (100)	6 (75.0)
Severe anemia^b^	28 (35.9)	19 (38.0)	10 (40.0)	11 (40.7)	3 (42.9)	5 (35.7)	0 (0.0)	4 (50.0)
BWF	9 (11.5)	29 (58.0)	5 (20.0)	16 (59.3)	2 (28.6)	8 (57.1)	0 (0.0)	4 (50.0)
Transfused	36 (46.2)	26 (52.0)	13 (52.0)	16 (59.3)	4 (57.1)	5 (35.7)	0 (0.0)	4 (50.0)
Total,^c^ n	78	50	25	27	7	14	1	8

Data are presented as n (%) unless otherwise noted.Abbreviation: BWF, blackwater fever.

^a^All children with positive blood smear or rapid diagnostic test for malaria.

^b^Severe anemia (Hb <5.0 g/dL) with or without malaria parasitemia.

^c^Some patients had more than 1 diagnosis so column totals do not add up to 100%.

### Timing of Readmission

Overall, 42 of 128 (32.8%) of the readmissions occurred within 6 weeks of discharge from the hospital, while 79 of 128 (61.7%) of the readmissions occurred within 3 months. The timing of readmissions was not significantly different when stratified according to readmission diagnoses or study groups.

### Deaths

**Figure 2.**
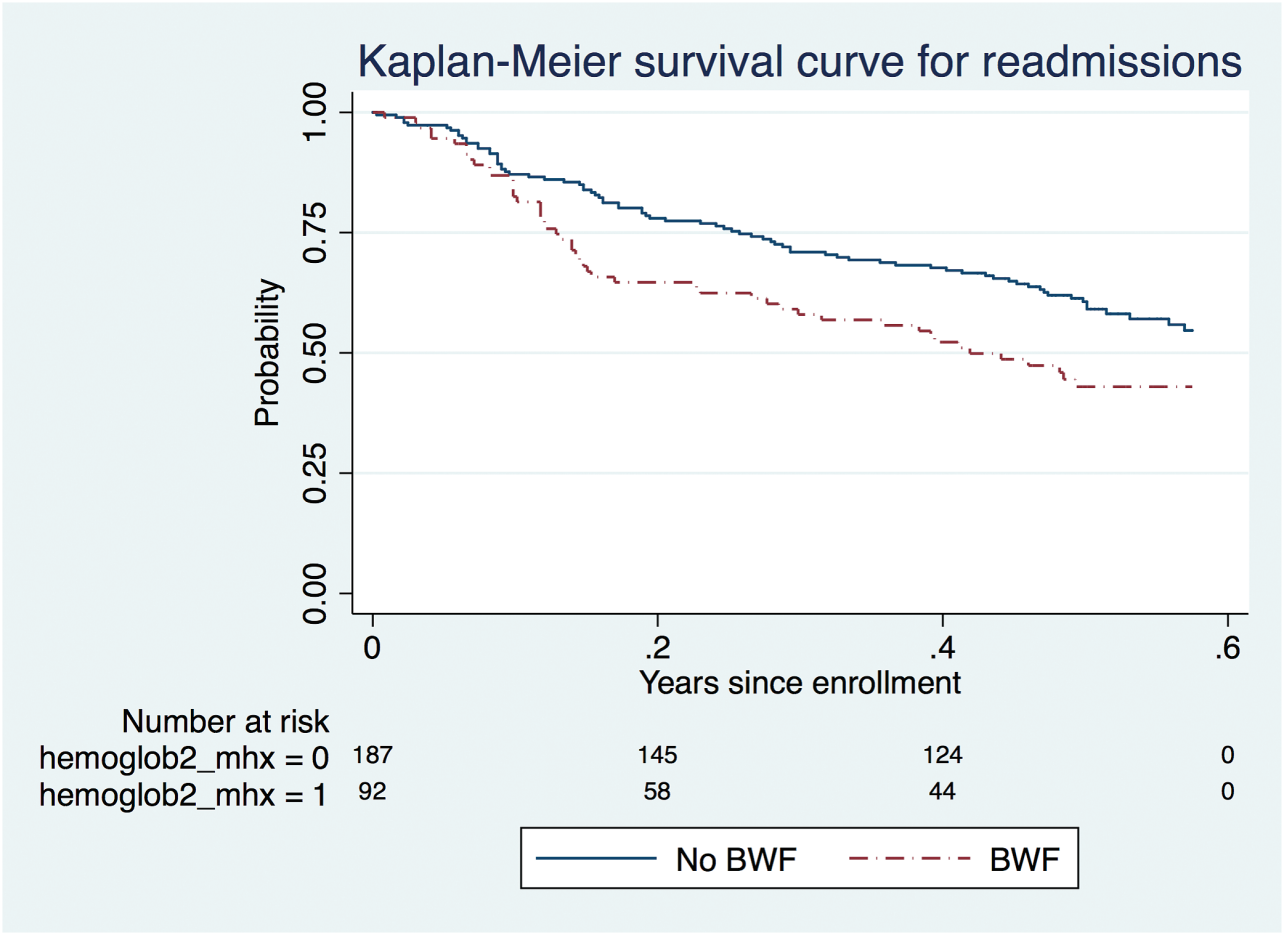
Time to readmission to the hospital in the 6-month postdischarge period for children with severe anemia with blackwater fever (BWF) and those without BWF. Abbreviation: hemoglobin2_mhx = 0, SA without BWF; hemoglobin2_mhx = 1, SA with BWF; SA, severe anemia.

**Figure 3.**
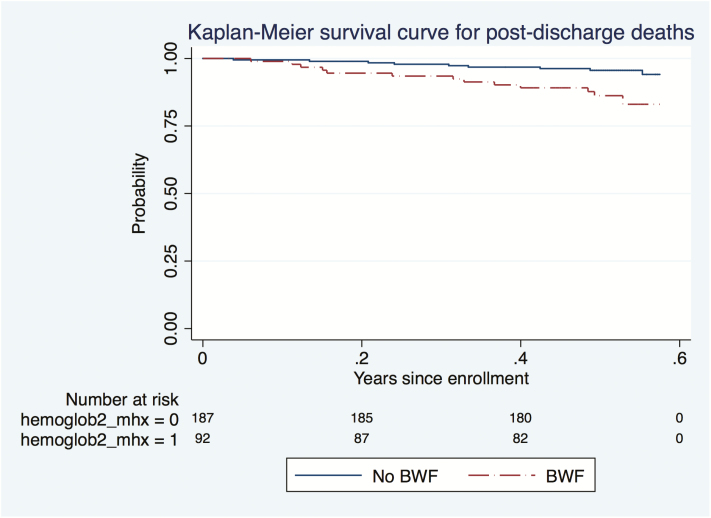
Time to death in the 6-month postdischarge period for children with severe anemia with blackwater fever (BWF) and those without BWF. Abbreviation: hemoglobin2_mhx = 0, SA without BWF; hemoglobin2_mhx = 1, SA with BWF; SA, severe anemia.

The majority (20/22; 90.9%) of the deaths occurred at home in the community, most of which were reportedly after an episode of acute febrile illness. The 2 children who died in the hospital were in the BWF group and in both cases severe malarial anemia was the reason for the readmission. In unadjusted analysis, children with SA with BWF had a statistically significantly shorter time to first readmission and death than children in the no-BWF group (Figures 2 and 3).

### Risk of Readmission and Deaths

After adjusting for age, sex, nutritional status, and parasitemia, children with SA with BWF had a significantly higher risk of readmissions (HR, 1.68; 95% CI, 1.1–2.5) and a greater risk of death (HR, 3.37; 95% CI, 1.3–8.5) compared with those without BWF (Table 4). However, there was no increased risk of outpatient visits in the children with SA with BWF compared with those without BWF.

**Table 4.** Hazard Ratios for Outpatient Clinic Visits, Postdischarge Readmissions, or Deaths Among 279 Children With Severe Anemia With and Without Blackwater Fever at Jinja Regional Referral Hospital

	Crude HR (95% CI)	*P*	Adjusted^a^ HR (95% CI)	*P*
Outpatient clinic visits				
No BWF (n = 187)	1.0 (ref)		…	
BWF (n = 92)	1.45 (.9, 2.4)	.138	1.46 (.9, 2.5)	.156
Readmissions				
No BWF (n = 187)	1.0 (ref)		…	
BWF (n = 92)	1.73 (1.2, 2.5)	.003	1.68 (1.1, 2.5)	.008
Deaths				
No BWF (n = 187)	1.0 (ref)		…	
BWF (n = 92)	3.20 (1.4, 7.5)	.007	3.37 (1.3, 8.5)	.010

Abbreviations: BWF, blackwater fever; CI, confidence interval; HR, hazard ratio; ref, reference.

^a^Adjusted for age, sex, stunting, and parasitemia.

## DISCUSSION

We followed up a cohort of children with SA for 6 months after discharge from the hospital. Nearly half (45.9%) the children with SA were readmitted after discharge, which is much higher than postdischarge rates of 9.4% [9] and 17.2% [7] previously reported. Disturbingly, there was a high rate of multiple readmissions for all children with SA, especially in children with SA associated with BWF, where half (50%) had multiple readmissions. The overall postdischarge mortality of 7.5% found in this study was only slightly lower than the 13% reported in Kenyan and Malawian studies, which were conducted at a time of increasing malarial resistance to chloroquine [7, 8]. Overall, our study found a high burden of postdischarge morbidity in children with SA, particularly those with BWF. Children with SA with BWF had a 68% increased risk of readmission and were more than 3 times more likely to die in the 6-month postdischarge period than children with SA without BWF.

The overall rate of readmissions and deaths observed in SA children in this study is of public health concern. The readmission rate of 45.9%, is 4 times higher than 9.4%, which our group previously reported for children with severe malarial anemia admitted at Mulago hospital. Given that there have been no major changes in malaria treatment guidelines or admission criteria in the country, the likely explanation for disparity in the findings is the higher malaria transmission at Jinja RRH [24]. The high burden of malaria in this part of the country is associated with a correspondingly high burden of SA and BWF. In the Fluid Expansion As Supportive Therapy trial [20], the prevalence of SA and BWF at sites in eastern Uganda was approximately 2 times higher than that in Mulago: Soroti (SA, 43%; BWF, 22%) compared with Mulago (SA, 23%; BWF, 6%). Therefore, exposure of children with SA to repeat malarial attacks at a vulnerable period after discharge may be a key driver of the high readmission rates observed in this study. It is possible that the high burden of SA is compounded by other factors such as underlying poverty/malnutrition in this mainly rural population and other underlying genetic factors. Ongoing studies in the Jinja area have also noted similar high rates of admissions and readmissions. Further studies are therefore needed to establish the reasons for the high disease burden in this region. In the meantime, efforts are urgently therefore needed to reduce the burden of malaria in this population.

For children with BWF, epidemiological studies suggest that the interaction between host response to repeated malarial attacks, use of antimalarials, and possibly glucose 6 phosphate dehydrogenase deficiency are triggers for episodes of BWF [13, 26]. Blackwater fever is characterized by episodes of repeated acute hemolysis requiring hospitalizations for transfusions. In this study approximately 60% of children with BWF reported a history of transfusions prior to enrollment and a similar percentage required multiple transfusions at the time of enrollment hospitalization. Recent episodes of BWF in eastern Uganda were found to be triggered by the interaction between *P. falciparum* malaria and increased use of artemisinin derivatives [12]. Acute renal failure is thought to be the cause of death in children with SA with BWF [22, 26]. We were unable to ascertain whether children in this study died of renal failure since most of the deaths occurred in the community.

Irrespective of the associated diagnoses, malaria was the most common reason for readmission, accounting for more than 70% of the diagnoses at readmission. This suggests that, for children with SA, efforts should be focused on preventing exposure to malaria in the immediate postdischarge period. Although, overall, approximately 60% of the readmissions occurred in the first 3 months after discharge, readmissions due to malaria were spread over the entire 6-month follow-up period. Postdischarge malarial prophylaxis may therefore be required for at least 6 months after discharge. Phiri et al [27] showed that for children with severe malarial anemia, provision of malarial chemoprophylaxis in the first 3 months was associated with a 31% reduction in morbidity and mortality. A larger study is currently underway in Kenya and Uganda to determine whether malarial chemoprophylaxis will be effective and safe in reducing postdischarge mortality in children with SA. Another large clinical trial that examined a number of treatment strategies, including micronutrient supplements to reduce postdischarge morbidities in children with SA, has recently been completed [28]. The strategies included giving liberal amounts of blood transfusion, followed by postdischarge cotrimoxazole prophylaxis and or multivitamin-multimineral supplementation [28].

It is interesting to note that in our cohort only 2 children died in the hospital out of 213 episodes of hospitalizations. The majority of the deaths in this study occurred at home before the caretakers could bring the children back to the hospital. Postdischarge mortality was highest (14.1%) among children with SA with BWF, a finding similar to the 28-day mortality of 12% in children with BWF reported earlier in eastern Uganda [12]. Overall, these findings suggest that once the children made it to the hospital, they were more likely to be treated and to survive. This suggests that the inability to access urgent care may have been the underlying factor in postdischarge survival of these children.

The study had a number of limitations. During the follow-up period, approximately 12% of children with SA who were classified in the no-BWF group presented with features of BWF during the first readmission. It is therefore possible that these children developed BWF in the course of the study. Due to the paroxysmal nature of the condition, it was not possible to predict, during admission, which children would later develop features of BWF [12]. It is therefore possible that the true burden of postdischarge morbidity in children with SA with BWF is even higher than what was found in this study.

The study was conducted in only 1 site in eastern Uganda, so the findings may be subject to particular genetic and epidemiological factors unique to that area. For children with BWF, we did not perform urine studies to confirm hemoglobinuria or test for glucose 6 phosphate dehydrogenase deficiency, which is thought to be an important risk factor for BWF [26]. Nonetheless, our study was pragmatic and was based on the diagnostic capabilities used in routine practice in a low-resource setting. This study therefore highlights the burden of and risk factors for the postdischarge outcomes for a clinical episode of SA in a malaria-endemic setting.

In conclusion, we found that children with SA in Jinja RRH have a high frequency of postdischarge readmission and death. Children with SA with BWF, in particular, had a poor postdischarge outcome, with increased risk of readmission and death. There is a need for implementation of postdischarge malaria chemoprophylaxis for children with SA in malaria-endemic areas. Future studies should focus on understanding the pathophysiological mechanisms of BWF in children living in malaria-endemic areas.

## Notes

***Authors’ contributions.*** R. O. O., J. K. T., C. C. J., and C. K. were involved in the design of the study. R. O. O. conducted the study with the help of A. W. and H. N. R. O. O. wrote the first draft of the paper. All authors participated in manuscript writing and approved the final manuscript.

***Acknowledgments.*** The authors thank Jinja Regional Referral Hospital and Global Health Uganda for the permission to conduct this study in their facilities, all staff at these centers for their contribution to this work, and Gloria Kyarisiima, Gorretti Kemigisha, and Aliwuya Sadat for dedication in collecting and compiling the data.

***Disclaimer.*** The funders had no role in the design of the study; in the collection, analysis, and interpretation of the data; or in writing and preparation of the manuscript.

***Financial support.*** This work was supported by the National Institutes of Health (NURTURE; grant number D43TW010132), the Fogarty International Centre (grant number D43 NS078280), and the Research Council of Norway through the Global Health and Vaccination Programme (GLOBVAC; project number 234487).

***Potential conflicts of interest.*** The authors report no potential conflicts of interest. All authors have submitted the ICMJE Form for Disclosure of Potential Conflicts of Interest. Conflicts that the editors consider relevant to the content of the manuscript have been disclosed.
